# Neonatal warming devices: What can be recommended for low-resource settings when skin-to-skin care is not feasible?

**DOI:** 10.3389/fped.2023.1171258

**Published:** 2023-04-25

**Authors:** Michiko Kyokan, Flavia Rosa-Mangeret, Matthieu Gani, Riccardo E. Pfister

**Affiliations:** ^1^ Institute of Global Health, University of Geneva, Geneva, Switzerland; ^2^ Department of Neonatology, Geneva University Hospitals and Geneva University, Geneva, Switzerland; ^3^ Essential Medical Devices Foundation, Lausanne, Switzerland

**Keywords:** neonate, thermal care, warming device, hypothermia, low-resource settings

## Abstract

Hypothermia occurs frequently among clinically unstable neonates who are not suitable to place in skin-to-skin care. This study aims to explore the existing evidence on the effectiveness, usability, and affordability of neonatal warming devices when skin-to-skin care is not feasible in low-resource settings. To explore existing data, we searched for (1) systematic reviews as well as randomised and quasi-randomised controlled trials comparing the effectiveness of radiant warmers, conductive warmers, or incubators among neonates, (2) neonatal thermal care guidelines for the use of warming devices in low-resource settings and (3) technical specification and resource requirement of warming devices which are available in the market and certified medical device by the US Food and Drug Administration or with a CE marking. Seven studies met the inclusion criteria, two were systematic reviews comparing radiant warmers vs. incubators and heated water-filled mattresses vs. incubators, and five were randomised controlled trials comparing conductive thermal mattresses with phase-change materials vs. radiant warmers and low-cost cardboard incubator vs. standard incubator. There was no significant difference in effectiveness between devices except radiant warmers caused a statistically significant increase in insensible water loss. Seven guidelines covering the use of neonatal warming devices show no consensus about the choice of warming methods for clinically unstable neonates. The main warming devices currently available and intended for low-resource settings are radiant warmers, incubators, and conductive warmers with advantages and limitations in terms of characteristics and resource requirements. Some devices require consumables which need to be considered when making a purchase decision. As effectiveness is comparable between devices, specific requirements according to patients' characteristics, technical specification, and context suitability must play a primary role in the selection and purchasing decision of warming devices. In the delivery room, a radiant warmer allows fast access during a short period and will benefit numerous neonates. In the neonatal unit, warming mattresses are low-cost, effective, and low-electricity consumption devices. Finally, incubators are required for very premature infants to control insensible water losses, mainly during the first one to two weeks of life, mostly in referral centres.

## 1. Introduction

2.4 million neonates die annually, 80% in sub-Saharan Africa and South Asia and 99% in low- and middle-income countries (1). Hypothermia has been widely regarded as a major contributor to neonatal mortality and morbidity (2, 3). Its high prevalence has been reported in low- and middle-income countries (4, 5). Premature neonates are at an increased risk of hypothermia, which has been associated with up to 80% of deaths in this group (6).

Neonates, particularly those with low birth weight, cannot maintain their body temperature without an appropriate thermal environment (7). Skin-to-skin care reduces the risk of hypothermia, and it is recommended by the World Health Organization (WHO) as an effective heat source for preterm or low birth weight neonates unless the neonate is in shock or needs resuscitation or mechanical ventilation (8). Other prevention measures exist such as plastic wraps and cap (9) but they are not sufficient in certain circumstances. Hypothermia is frequent in clinically unstable neonates who require resuscitation, respiratory support, or other interventions with a high risk of death (4) and it is often not feasible to place clinically unstable neonates in skin-to-skin care. In addition, data on the effectiveness of skin-to-skin care in this high-risk population is scarce (8, 10). Furthermore, parents and family members may not be able to provide continuous skin-to-skin care due to sickness, other competing activities and cultural norms (11). Skin-to-skin care is recommended continuously for 24 h a day, but uninterrupted skin-to-skin care is often not provided (10, 12, 13). Therefore, warming devices become indispensable to provide an optimal thermoneutral environment for neonates who are clinically unstable or whose parents or family members cannot provide continuous skin-to-skin care.

WHO recommends using radiant warmers or incubators for unstable neonates weighing 2000g or less, or for stable neonates below 2,000 g who cannot receive skin-to-skin care (14). In low-resource settings, however, these warming devices are often unavailable (15), either broken down, with little chance of being made functional again on site (16) or kept in storage due to missing parts, insufficient power-grid or lack of consumables (17).

Clear and concise clinical guidance for the use of warming devices, based on the best available evidence for healthcare workers and policymakers is necessary to reduce hypothermia-related neonatal morbidity and mortality in low-resource settings. Therefore, we aim to explore the existing evidence on the effectiveness, usability, and affordability of neonatal warming devices when skin-to-skin care is not feasible.

## 2. Methods

To explore existing data, we conducted a *rapid review* which synthesis evidence within a shorter timeframe than the systematic review process (18). This review includes: (1) a search for systematic reviews as well as randomised and quasi-randomised controlled trials (*rapid review*), which is complemented by (2) a search for neonatal thermal care guidelines for the use of warming devices in low-resource settings and (3) a search for specification and resource requirement of warming devices. Searches were conducted in May 2022. One author (MK) extracted all data, and it was checked by a second author (RP). The protocol has been registered at OSF registries (19).

### 2.1. A search for systematic reviews, randomised and quasi-randomised controlled trials (*rapid review*)

Study designs eligible for inclusion in the review comprised: systematic reviews with or without meta-analyses, randomised controlled trials (RCT), and quasi-randomised controlled trials. The search strategy was structured with the relevant terms as follows: (1) Population = neonates (age 0–28 days); and (2) Intervention = radiant warmers, conductive warmers, or incubators. We have opted not to limit the search only to a specific comparator or outcome, to maximise the number of results. The search was performed in the following databases: Pubmed, Embase, and Cochrane library, including the Database of Systematic Reviews and Cochrane Central Register of Controlled Trials. A full electronic search is provided in the Supplementary Material S1. To maximise the number of entries, we therefore opted not to restrict the search to low-income settings and a specific time frame. We chose to take a stepwise approach, emphasising systematic reviews first and then including randomised and quasi-randomised controlled trials (18). Studies already included in the systematic reviews were not eligible for inclusion in this extension of the review.

The following studies were excluded from the review: non-systematic reviews, discussion papers, letters, and editorials, qualitative studies, cohort studies, case studies, case series, before and after studies and other lower quality designs, animal studies, abstracts and studies not available or obtainable in full text, unpublished material, and publications in languages other than English or French.

Retrieved records were uploaded into EPPI-Reviewer (20) for screening. MK and RP conducted a pilot exercise with the same 92 abstracts, 10% of all identified articles, to calibrate and test the screening process. Then, MK checked the titles and abstracts of identified studies according to the above selection criteria and categorised them as: included, not included and unsure. For those papers in the unsure category, MK checked the full text and re-categorised as above after discussion with RP. Full-text copies of potentially relevant studies were obtained, and their eligibility for inclusion was assessed.

The methodological quality of included studies was assessed using AMSTAR2: A Measurement Tool to Assess Reviews (21) for systematic reviews and the Cochrane Risk of Bias Tool for RCTs (22). A Risk of Bias table is provided in the Supplementary Material S2. The authors used the study quality assessment to interpret the study results in this review.

For each study, the following information was extracted by MK using Microsoft Excel: the first author, year, study design, setting, sample, intervention, types of measures, risk of bias assessment and findings. RP then checked the data extraction process. Finally, a narrative synthesis was undertaken as well as a descriptive summary with data tables of the literature (Table 1).

**Table 1:** Summary of included studies.

	Interventions	Gestational ages	Birthweights	Ages at entry	Country	No. of studies	No. of participants	Conclusions
**Systematic reviews**								
Flenady and Woodgate, 2003	Radiant warmer vs. incubator	28–32 weeks^*^	1,100–1,600 g^*^	Majority of infants older than seven days	Developed countries	8	165	Radiant warmers resulted in increased insensible water loss compared to incubators needing consideration when calculating their daily fluid requirements. No sufficient evidence was provided concerning the effects on important outcomes, such as oxygen consumption, weight gain, morbidities and mortality to guide clinical practice.
Gray and Flednady, 2011	Heated water-filled mattress vs. incubator	29–35 weeks^*^	1,088–1,621 g^*^	*N* = 103 (42%) < 7 days *N* = 120 (58%) 11–20 days	Australia Sweden Ethiopia Turkey	5	223	Cot-nursing using a heated water-filled mattress had similar effectiveness to incubator care on temperature control and weight gain. A trend towards reduced mortality before hospital discharge was found among preterm infants who were allocated to water-filled mattresses.
**Randomised control trials**								
Meyer and Bold, 2007	Radiant warmer vs. incubator	26 weeks	870–902 g	Once stable after delivery	New Zealand	–	62	The study did not find significant differences in admission temperature for infants occlusively wrapped and transported either *via* radiant warmer or incubator. There were no significant relationships between the secondary outcomes and warming devices or admission temperatures.
Bhat et al. 2015	Conductive thermal mattress with phase-change materials vs. radiant warmer	35 weeks	1,931–1,959 g	6.5–7.5 days (mean)	India	–	128	Short-term use of conductive thermal mattresses compared with radiant warmers and other warming modes was non-inferior to radiant warmers and effective in maintaining body temperature. No adverse effects were reported.
Vijayan et al. 2020	Conductive thermal mattress with phase-change materials vs. radiant warmer	31 weeks	1,435–1,447 g	12 days (mean)	India	–	66	Conductive thermal mattresses were comparable to radiant warmers in thermoregulation of hospitalised stable preterm infants.
Chandrasekaran et al. 2021	Low-cost cardboard incubator vs. conventional incubator	30–35 weeks	1,220–2,120 g	2–16 days (mean)	India	–	96	Thermoregulation of stable preterm infants in the redesigned low-cost 200 USD cardboard incubator was non-inferior compared to the conventional incubator. Low-cost cardboard incubators could be a stop-gap measure in high-risk infants for up to a week until the a proper device becomes available.

^*^ Mean gestational ages and birth weights across the included studies.

### 2.2. A search for neonatal thermal care guidelines for the use of warming devices in low-resource settings

To identify up-to-date guidelines, multiple sources were scrutinised. First, we searched for neonatal care guidelines from WHO, the United Nations Children's Fund (UNICEF), and international organisations that provide neonatal care in hospital settings, such as Médecins Sans Frontières and Save the Children. Healthy Newborn Network and Newborn Essential Solutions and Technologies (NEST 360) were also included, as they have been facilitating the development of neonatal care medical equipment for low-resource settings. An internet search in the Google search engine using the keywords “neonates”, “thermal care”, “neonatal care”, “warming device”, “hypothermia”, “guideline”, and “protocol” was also conducted to identify other guidelines covering the use of warming devices in the neonate. The inclusion criteria specified guidelines in English that cover the use of warming devices in neonates from low- and lower-middle-income countries (23). Finally, the most recent iteration was selected if multiple guideline versions were identified from the same source. For each identified guideline, its development methodologies, key recommendations, and their strength of evidence were extracted and assessed using Microsoft excel spreadsheet by MK. The data extraction process was checked by RP.

### 2.3. A search for specification and resource requirements of warming devices

Multiple sources were used to identify warming devices' characteristics and resource requirements. First, we searched for medical device catalogues of WHO, UNICEF, Médecins Sans Frontières and NEST 360. An internet search in the Google search engine using the keywords' neonates', “warming devices”, “warmers”, and “hypothermia” was also conducted. The inclusion criteria specified data in English. For data identified on each warming device, key characteristics and resource requirements were extracted and assessed by MK. The data extraction process was checked by RP.

## 3. Results

### 3.1. A search for systematic reviews, randomised and quasi-randomised controlled trials (*rapid review*)

**Figure 1:**
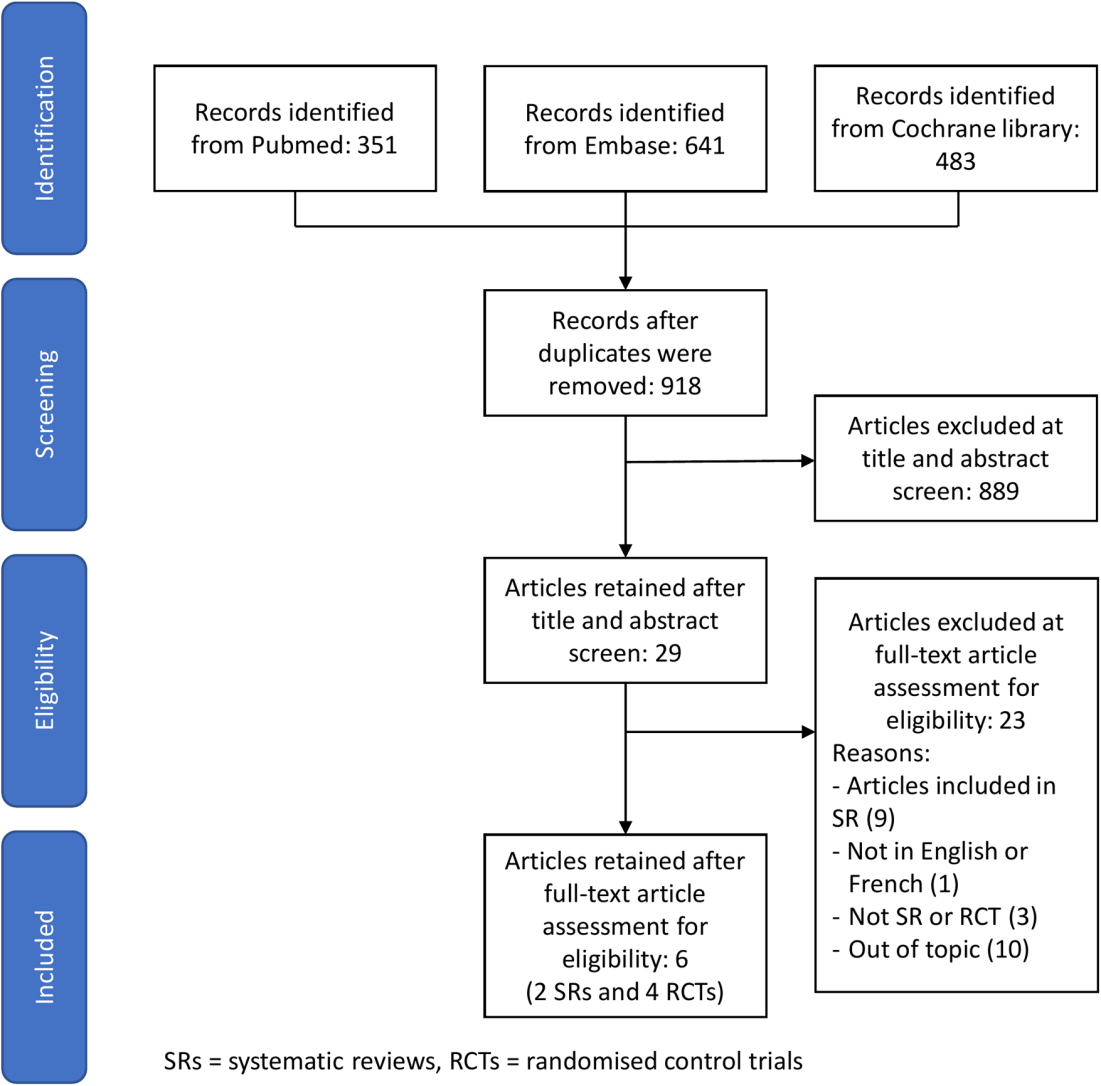
Study flow diagram.

29 studies were considered potentially eligible for inclusion in this review after removing duplicate studies and screening the title and abstract. Seven studies met the inclusion criteria, two were systematic reviews and five were RCTs. The selection process is shown in Figure 1. Study summaries are listed in Table 1.

#### 3.1.1. Radiant warmers vs. incubators

Flenady and Woodgate reviewed the effects of radiant warmers vs. incubators on neonatal fluid and electrolyte balance, morbidity and mortality (24). Eight RCTs comprehending 165 neonates were included in this systematic review. All studies enrolled preterm infants (mean gestational ages 28–32 weeks, mean birthweights 1,100–1,600 g across the trials). Most neonates were older than seven days when studied. The review concluded that radiant warmers caused a statistically significant increase in insensible water loss (mean difference 0.94 g/kg/day; 95% CI 0.47 to 1.41 g/kg/day). Due to a small number of participants, the effects on important clinical outcomes, such as oxygen consumption, weight gain, morbidities and mortality could not be adequately assessed.

An RCT from a high-income country conducted after the Flenady and Woodgate study, assessed the effects of radiant warmers compared to incubators in conjunction with an occlusive wrap immediately after birth (25). Their primary outcome was admission temperature in the neonatal unit. 62 neonates with a postmenstrual age ranging from 23 to 27 weeks and a birthweight ranging from 746 to 1,031 g were included. They concluded that the difference in admission temperature between these two warming devices in occlusively wrapped neonates was not statistically significant. In addition, most neonates achieved the target admission temperature in both devices.

#### 3.1.2. Heated water-filled mattresses vs. incubators

Gray and Flenady reviewed the effects of heated water-filled mattresses vs. incubators on temperature control and weight gain in preterm infants (26). Overall, five RCTs with a total of 223 neonates were included. Heated water-filled mattresses appeared to be as effective as incubators regarding temperature control and weight gain. In their meta-analysis, the authors found a reduced relative risk of mortality before hospital discharge allocated to water-filled mattresses that was not statistically significant (RR: 0.63; 95% CI: 0.3–1.34). This trend was related mainly to one trial undertaken in a neonatal care unit in Turkey where only one full-time nurse was available during the daytime and one single nurse on duty for more than one unit during night-time (27).

#### 3.1.3. Conductive thermal mattresses with phase-change materials (PCM) vs. radiant warmers

Two other RCTs not included in the systematic reviews compared the effects of conductive thermal mattresses with PCMs that provided four to six hours of heat after the PCM pouch was pre-heated to 37°C (28, 29). An electric heater was used to reach the desired temperature before the PCM pouch was placed inside a specially designed sleeping bag. Both RCTs were conducted in India. In the RCT on 128 late preterm infants conducted by Bhat et al., conductive thermal mattresses were non-inferior to radiant warmers with a higher axillary temperature by a mean of 0.14 ± 0.03°C (lower bound of 95% CI: 0.14–0.06°C) (28). The other RCT conducted by Vijayan et al. found no significant difference between groups of smaller preterm infants, neither in the proportion of out-of-range temperature events nor in physiological instability or mean weight gain (29). Neither study reported any adverse events, such as skin burns. However, both studies were conducted in stable preterm infants only and limited by the short duration of equipment comparisons of four hours and 24 h, respectively.

#### 3.1.4. Low-cost cardboard incubator vs. standard incubator

One RCT assessed a prototype incubator comprised of a disposable cardboard chamber and a modular heating unit priced at 200 USD (30). The servo-controlled heater, air temperature sensor and skin temperature thermistor sensor were identical to the ones used in the single wall comparison incubator and complied with IEC60601-1 standards. 96 stable preterm infants were enrolled in this study which lasted 48 h. Overall, the low-cost incubator along with skin-to-skin care was found to be non-inferior to the standard single-wall incubator and without adverse events. Mean skin and axillary temperatures were within the non-inferiority limits and failed thermoregulation, defined as abnormal axillary temperature <36.5°C or >37.5°C for longer than 30 continuous minutes, never occurred.

### 3.2. Neonatal thermal care guidelines for the use of warming devices in low-resource settings

Seven guidelines covering the use of neonatal warming devices that met the selection criteria outlined in the methodology were identified. These included three guidelines from WHO (14, 31, 32) and five guidelines from the Ministries of Health in lower-middle-income countries: Cambodia (33), Egypt (34), Eswatini (35), Papua New Guinea (36), and Palestine (37). The most acknowledged neonatal thermal care guideline, which still remains the most frequently cited reference today, was first published by WHO in 1993 (38) and updated in 1997 (31). Although the guideline might be outdated in some aspects (39), we chose to include it, because no more-recent thermal care guidelines with the same details and coverage have been published by WHO since. Other included guidelines in this review are general neonatal care guidelines with a chapter on thermoregulation.

We provide an extensive summary of the key recommendations made by each guideline regarding processes to keep neonates warm and rewarm hypothermic neonates in the Supplementary Material S3. Overall, the strength of evidence appears to be limited, as only WHO Recommendations on Newborn Health (14) and Paediatrics for Doctors of Papua New Guinea (36) are partially based on systematic reviews of a very small number of trials, or one single RCT, thus finally on a small overall number of neonates. Other guidelines are almost exclusively based on earlier published guidelines and protocols. The following paragraphs summarise available thermal care recommendations in different clinical and environmental conditions.

#### 3.2.1. Keeping neonates warm at birth and during neonatal resuscitation

For all healthy newborns at birth, WHO (14, 31, 32) and numerous guidelines (33–37) recommend immediate skin-to-skin care. A review of this recommended method is outside the scope of this review. For neonatal resuscitation or during procedures where the mother cannot directly provide warmth, WHO's Thermal Protection of the Newborn (31), recommends radiant warmers and proposes to replace radiant warmers with alternative means as soon as possible (31, 40). In consensus, three other guidelines also recommend radiant warmers for resuscitation (33, 34, 37).

#### 3.2.2. Keeping clinically stable neonates warm

A consensus among analysed guidelines favours skin-to-skin care to keep clinically stable neonates warm. WHO's Recommendations on Newborn Health include low birth weight neonates weighing >1,200 g without complications for these indications (14), and the Cambodian Nursing Manual for Neonatal Care Unit (33) specifies “breathing spontaneously without additional oxygen” as a condition for skin-to-skin care.

#### 3.2.3. Keeping clinically unstable neonates warm

For clinically unstable neonates, or stable neonates who cannot be given skin-to-skin care, published guidelines show some discrepancies. WHO's Recommendations on Newborn Health (14) and the Eswatini Neonatal Care Clinical Guidelines (35) recommend radiant warmers or incubators for unstable low birth weight neonates. The Palestine National Neonatal Protocol (37) also recommends both devices but specifies for unstable neonates <1,500 g to use incubators. In contrast, Paediatrics for Doctors of Papua New Guinea recommends neither radiant warmers nor incubators, but heated water-filled mattresses, warm rooms at 27–30°C, or electric blankets (36). They argue that when skin-to-skin is unfeasible for low birth weight neonates, heated water-filled mattresses are more affordable and safer than incubators.

#### 3.2.4. Rewarming hypothermic neonates

Some guidelines specifically recommend rewarming methods for hypothermic neonates, again with diverging views. For example, WHO's Thermal Protection of the Newborn (31) recommends skin-to-skin care in a warm room of 25°C at least for mild hypothermia (36.0–36.4°C), radiant warmers or incubators for moderate hypothermia (32.0–35.9°C) and incubators for severe hypothermia (<32.0°C). The broader subsequent WHO's Managing Newborn Problems (32) guideline recommends four rewarming methods for temperatures <36.5°C, stating the high priority for those <32°C: (1) skin-to-skin and (2) warm room ≥26°C for stable neonates without life-threatening conditions, (3) radiant warmers for neonates weighing ≥1,500 g and (4) incubators for neonates weighing <1,500 g. The Cambodian Nursing Manual for Neonatal Care Unit (33) similarly recommends radiant warmers as the first intention for immediate rewarming of hypothermic neonates <34.9°C or in case of prolonged hypothermia. In contrast, the Eswatini Neonatal Care Clinical Guidelines (35) and the Palestine National Neonatal Protocol (37) recommend always rewarming neonates with incubators. Their preference for incubators over radiant warmers is argued based upon having a “better” control of the temperature.

#### 3.2.5. Alternative methods when no devices are available

For emergencies, when no other device or method is available, WHO's Thermal Protection of the Newborn (31) mentions alternative low-cost, do-it-yourself strategies, such as incandescence light bulbs, hot water bottles or heated bricks which should be removed before placing a neonate for safety. In addition, the Cambodian Nursing Manual for Neonatal Care Unit (33) also recommends placing warm bottles covered with cloth around the neonate to avoid hypothermia in case of need.

### 3.3. A search for specification and resource requirements of warming devices

The main warming devices currently available and intended for low-resource settings are radiant warmers, incubators, and conductive warmers (16). Table 2 summarises their main characteristics with advantages and limitations.

**Table 2:** Summary characteristics of warming devices for neonates^1^ (16, 41–47).

		Power	Accessibility/Visibility	Risk of overheating or burn	Insensible water loss	Humidity control	Maintenance and repair	Cleaning	Training needs	Purchase price	Consumables and others	Comments
Radiant warmer		Main (high, 600 Watts∼)	Good/Good	High	High	Not available	Medium	Easy	Medium	High	Skin probe (optional)	Practical device for procedures such as resuscitation. Ideal to place at least one device in a delivery room for naked babies soon after birth. Use devices with caution for neonates <30 weeks gestation and < 2 weeks of age because of high insensible water loss. Due to risk of burn from intense radiant heat, uncontrolled prolonged use to be avoided.
Incubator		Main^2^ (high, 100–700 Watts)	Medium/Medium	Medium	Low	Available	Difficult	Difficult	High for both operation and maintenance	Very high	- Skin probe - Fan, filter, motor, humidity chamber - Long training, maintenance, and cleaning time	Expensive device to purchase, run and maintain. Ideal for VLBW babies. Many parts need dissembling and assembling for cleaning between patients and maintenance.
Conductive warmer	Mattress	Main^2^ (low, 10 Watts∼)	Limited/Limited^3^	Low	Low	Not available	Medium	Medium	Low	High	Skin probe (optional)	Due to its ease of use, ideal for facilities with limited qualified staff. Conductive heat may not be sufficiently intense for rewarming at the required speed.
	PCM	Main, boil, autoclave, microwave	Limited/Limited^3^	Low	Low	Not available	Easy	Medium	Low	Low	Warming pad	Pre-heating is necessary before use. The warming pad lasts 4–6 h, then needs pre-heated again (needs planning). Ideal for transportation when skin-to-skin is not feasible. Non-electric types are ideal where there is no or unreliable electricity supply. Periodic (100 times or 6 months) replacement of warming pad increases the cost of ownership.

^1^ Certified medical device by the US Food and Drug Administration (FDA) or with a CE marking only.

^2^ Some devices have a battery option.

^3^ Removing the baby wrap provides better accessibility and visibility but increases heat loss.

#### 3.3.1. Radiant warmers

Radiant warmers use overhead heat sources and may use a feedback loop to servo-control the neonate's temperature (48). In addition, good accessibility to and visibility of the neonate make these devices ideal when continuous observation and fast access to the naked baby is required, such as during neonatal resuscitation or invasive procedures (49).

One of the unfavourable effects of radiant warmers is the increased insensible water loss through evaporation. Radiant warmers, therefore, need to be used with caution, especially for neonates <30 weeks gestation and <two weeks of age when the skin is still immature and allows significant insensible water losses (50). Radiant warmers also risk causing skin burns due to excessive radiant heat, either when a heat source is too close to the neonate or the heat output is too high (51). Thus, close monitoring of neonates and the individual radiant warmer's settings is crucial. Another downside of radiant warmers is the high electric power requirements without autonomy by a battery (52).

#### 3.3.2. Incubators

Incubators create a microclimate where temperature and humidity (and for some devices, oxygen) can be regulated individually. Incubators draw room air *via* a filter and warm it with a heating element (7). The temperature control is regulated with a thermostat based on air or patient skin temperature. The enclosure also allows some devices to control humidity. High humidity is advantageous for very premature infants or may even be essential for extremely premature infants who experience very high insensible water loss due to their immature skin, particularly during their first weeks of life (53). Warming devices other than incubators cannot provide controlled humidity.

Several limitations of incubators hamper their use in low-resource settings. First, incubators are expensive to purchase and often require consumables such as skin probes for temperature monitoring and replacement parts such as air filters and sterile water if humidity is used, significantly increases the total cost of ownership in the long run (54). Second, to correctly operate an incubator, healthcare professionals need to understand the physiological needs of the neonate and the technical functions of the incubator, as well as their interaction, thus demanding close monitoring (55). For instance, the incubator's temperature drops inevitably whenever the incubator's doors are opened to access the neonate (56), mainly when using high humidity settings.

In addition, maintenance and cleaning require technical competence and considerable human resources for dissembling and assembling between patients. Improper maintenance and cleaning increase the risk of malfunction and infection (57, 58). Furthermore, the common practice in low-resource settings of placing multiple neonates in a single incubator increases the risk of cross-infections (55).

It must also be noted, particularly for warm environments, that incubators cannot cool. The lowest running temperature is 2–3 degrees Celsius higher than the environmental room temperature due to the generated running temperature (7). Therefore, a set temperature may not be reachable when low temperatures are required for larger babies. High ambient temperatures are typical in tropical countries but also in controlled heated neonatal care units.

Finally, a concern about incubators may be the constant noise of the fan-operated airflow. Even though the American Academy of Pediatrics recommends avoiding sound levels above 45 dB in neonatal care units, some studies demonstrated noise within incubators exceeded these recommendations frequently (59, 60), disrupting sleep cycles (61) and possibly influencing hearing development.

#### 3.3.3. Conductive warmers

Conductive warmers transfer heat from equipment below or around the patient (62). Skin-to-skin care is *de facto* a conductive heat transfer from the mother to the baby and is discussed separately in this review. The power consumption of conductive warmers is generally much lower than radiant warmers and incubators (41). Two types of conductive warming equipment are often considered separately: (1) stationary warmers (63) and (2) transportable cocoon-type warmers (29, 64). Stationary devices are usually operated on the main electric grid. Still, some can be used with one battery for a limited time, allowing continued operation in the event of power cuts (63).

Transportable cocoon-type warmers mostly use a PCM which releases energy at phase transition temperatures depending on the material chosen and can provide heat for up to 4–6 h (29, 64). PCMs can be used to maintain a constant temperature as they absorb and release large amounts of latent heat when they change their physical state between liquid and solid (65). As long as the PCM is neither fully solid nor liquid, its specific transition temperature remains constant. The latent heat absorbed by the PCM can be stored therein and act as thermal storage. Before use, PCMs need pre-heating by various means such as electricity or boiling water, and autoclaving may be possible for some devices to increase hygiene (64).

## 4. Discussion

The results of this review showed only limited evidence and consensus about the best warming method for neonates who are clinically unstable or whose parents or families cannot provide skin-to-skin care. Limitations of this review include small numbers of identified studies; small sample sizes; and searches for publications in English or French. Most studies were conducted in high-income countries classified by the World Bank, except for Ethiopia, Turkey and India (24, 26, 29, 30, 66). In addition, the studies' participants were mostly limited to neonates without medical complications and those older than seven days. It has to be also noted that the strength of evidence appears to be limited for identified guidelines which are mostly based on earlier published guidelines and protocols. Despite these limitations, there is reasonable evidence to conclude that there is no significant difference between radiant warmers, incubators, and conductive thermal mattresses in terms of warming effectiveness by a comprehensive literature search for both published and unpublished studies by the authors.

Radiant warmers provide immediate access to the patient. It is desirable for a delivery room to have a radiant warmer available, as some form of neonatal resuscitation occurs as often as one in ten babies (67), and fast and accurate resuscitation manoeuvres may not be feasible in skin-to-skin care. A potential increase in insensible water loss under radiant warmers needs to be considered for daily fluid requirements of premature and low birth weight neonates (24).

Based on the available evidence, neonates with birth weight <1,500 g during the first one to two weeks of life may benefit from the humidity feature of incubators for prolonged thermal care. The care of such infants is generally limited to high level neonatal units. The use of incubators possibly does more harm than good in healthcare facilities where the qualification level of the staff is basic and there are too few nurses trying to care for too many patients (31). The recommendation of the Ministry of Health of Papua New Guinea to use heated water-filled mattresses instead of radiant warmers and incubators follows current evidence and is adapted to the local context (27). From the perspective of risk-benefit and value-for-money analysis, we believe that existing incubators may not be the best choice where monetary and human resources are limited and should be reserved for high-level neonatal units that care for extreme premature neonates specifically needing high humidity.

Conductive thermal mattresses present a promising low-cost option without compromising effectiveness in maintaining neonatal body temperature, as demonstrated by a systematic review by Gray and Flenady (26). Its ease of use and low electric consumption make the device more suitable than radiant warmers and incubators in neonatal care units in low-resource settings (27). Further improvement has been introduced with heat storing PCMs in these mattresses. Transportable cocoon-type warmers with PCMs appear ideal for transportation as they are mobile and do not require electricity for several hours. Two RCTs in neonates older than seven days confirmed the effectiveness of these warmers with PCMs compared to radiant warmers (28, 29). Although their effectiveness during the early postnatal period may be debatable, there is no apparent reason for a significant discrepancy in effectiveness in earlier use. However, pre-heating requires a workforce and time, thus, planning and organisation are necessary for use longer than 4–6 h. In addition, PCMs have a single set temperature that might not be ideal for all neonates. An unchangeable constant conductive warming temperature may not be sufficient for rewarming hypothermic neonates or for specific patient conditions, such as naked neonates during medical interventions. Therefore, conductive warmers are usually not intended for critically ill neonates (68). Technical improvements combining servo-controlled reheating and/or combination with other modes of thermal care is promising and need further research and development. None of the present guidelines, including the WHO guidelines, mention conductive thermal mattresses with PCMs. We believe they need to be included in recommendations for use in low-resource settings.

As effectiveness is comparable between devices, specific requirements such as fast access, rewarming of established hypothermia or minimisation of insensible water losses, context suitability based on patients' characteristics, facility admission volume, financial and human resources, and technical specification must play a primary role in the purchase decision. The health facilities must meet infrastructural requirements such as space, power supply and security, particularly considering the consumption of multiple devices been added up. The risk of power cuts can be mitigated by devices with a battery option, whether electrical or by PCMs. It is also necessary to consider the total cost of ownership over purchase price, thus including operating and maintenance costs, as well as training and consumables over the device's lifespan which are summarised in Table 2. Overall, the total cost of ownership of incubators is high compared to radiant warmers and conductive warmers. Finally, the cost and means of disposing of a device, when the manufacturer has ended support, and replacement or repair parts for the device are no longer available, or if the device has broken down and cannot be fixed, must be considered.

## 5. Conclusions

Our review provides the best available evidence-based guidance for healthcare workers and stakeholders to choose appropriate warming devices for clinically unstable neonates whose parents or family members cannot offer skin-to-skin care. It should also help policymakers and donors to plan appropriate procurement of warming devices for low-resource settings. As there is no one-size-fits-all approach, each health facility needs to make the proper choice of devices according to their patients' characteristics and resources. Skin-to-skin care is clearly recommended for most neonates. In the delivery room, a radiant warmer allows fast access during a short period and will benefit numerous neonates. In the neonatal unit, warming mattresses are low-cost, effective, and low-electricity consumption devices. Finally, incubators are required for very premature infants to control insensible water losses, mainly during the first one to two weeks of life, mostly in referral centres. Further technical developments should target safety issues, energy efficiency and low total cost of ownership. Additional RCTs are required to assess the best device for clinically unstable neonates in the early neonatal period in low-resource settings.
