# Supplementary material for: Neonatal warming devices: What can be recommended for low-resource settings when skin-to-skin care is not feasible?
Source: Front Pediatr. 2023 Apr 25;11:1171258. doi: 10.3389/fped.2023.1171258 (PMC10167045; doi:10.3389/fped.2023.1171258)
Supplement: Supplementary file 1 [file Table1.docx]

Supplementary Material

Neonatal warming devices: what can be recommended for low-resource settings when skin-to-skin care is not feasible?

**Michiko Kyokan^1*^, Flavia Rosa-Mangeret^1,2*^, Matthieu Gani^3^, Riccardo E Pfister^1,2^**

^1^Institute of Global Health, University of Geneva, Geneva, Switzerland.

^2^Department of Neonatology, Geneva University Hospitals and Geneva University, Geneva, Switzerland.

^3^ Essential Medical Devices Foundation, Lausanne, Switzerland.

*** Correspondence:**Michiko Kyokan
michiko.kyokan@etu.unige.ch

**Supplementary material 1. Search strategy**

**Supplementary material 2. Risk of Bias table**

**Supplementary material 3. Guideline comparison for use of warming devices (key recommendations)**

**Supplementary material 1. Search strategy**

Pubmed

("infant, newborn"[MeSH Terms] OR "newborn"[Title/Abstract] OR "neonat*"[Title/Abstract]) AND ("incubators, infant"[MeSH Terms] OR "incubator*"[Title/Abstract] OR "infant incubator*"[Title/Abstract] OR "neonatal incubator*"[Title/Abstract] OR "newborn incubator*"[Title/Abstract] OR "infant radiant warmer*"[Title/Abstract] OR "radiant heate*"[Title/Abstract] OR "radiant warmer*"[Title/Abstract] OR "warming bed*"[Title/Abstract] OR "warming mattress*"[Title/Abstract] OR "heating mattress*"[Title/Abstract] OR "warming device*"[Title/Abstract] OR "heated water-filled mattress*"[Title/Abstract] OR "thermoregulation"[Title/Abstract] OR "warmer*"[Title/Abstract] OR "heater*"[Title/Abstract] OR"cot-nurs*"[Title/Abstract] OR "cot"[Title/Abstract]) AND ("Systematic Reviews as Topic"[Mesh] OR "Systematic Review"[Publication Type] OR "Randomized Controlled Trial"[Publication Type] OR "Randomized Controlled Trials as Topic"[Mesh] OR "Meta-Analysis"[Publication Type] OR "Meta-Analysis as Topic"[Mesh] OR "systematic"[Filter] OR "meta-analysis"[Publication Type] OR "meta-analysis as topic"[MeSH Terms] OR "meta analy*"[Text Word] OR "metanaly*"[Text Word] OR "metaanaly*"[Text Word] OR "met analy*"[Text Word] OR "systematic review*"[Title/Abstract] OR "randomized controlled trial"[Publication Type] OR "randomized controlled trials as topic"[MeSH Terms] OR "random allocation"[MeSH Terms] OR "double-blind method"[MeSH Terms] OR "single-blind method"[MeSH Terms] OR "random*"[Text Word] OR "Placebos"[MeSH Terms] OR "placebo"[Title/Abstract]) NOT ("Animals"[MeSH Terms] NOT "Humans"[MeSH])

Embase

('newborn'/exp OR newborn:ab,ti OR neonat*:ab,ti) AND ('neonatal incubator'/exp OR incubator*:ab,ti OR 'infant incubator*':ab,ti OR 'neonatal incubator*':ab,ti OR 'newborn incubator*':ab,ti OR 'infant radiant warmer*':ab,ti OR 'radiant heate*':ab,ti OR 'radiant warmer*':ab,ti OR 'warming bed*':ab,ti OR 'warming mattress*':ab,ti OR 'heating mattress*':ab,ti OR 'warming device*':ab,ti OR 'heated water-filled mattress*':ab,ti OR thermoregulation:ab,ti OR warmer*:ab,ti OR heater*:ab,ti OR 'cot nurs*':ab,ti OR 'cot':ab,ti) AND ('review'/exp OR 'systematic review'/de OR 'systematic review (topic)'/exp OR ((literature NEAR/3 review*):ab,ti) OR ((systematic* NEAR/2 review*):ab,ti) OR 'meta analysis'/de OR 'meta analysis (topic)'/de OR 'meta$anal*':ab,ti OR 'meta-anal*':ab,ti OR 'metaanal*':ab,ti OR 'randomized controlled trial'/de OR 'randomization'/de OR 'single blind procedure'/de OR 'double blind procedure'/de OR 'crossover procedure'/de OR 'placebo'/de OR 'prospective study'/de OR (('randomi?ed controlled' NEXT/1 trial*):ab,ti) OR rct:ab,ti OR 'randomly allocated':ab,ti OR 'allocated randomly':ab,ti OR 'random allocation':ab,ti OR randomized:ti,ab OR placebo:ti,ab OR randomly:ti,ab OR ((allocated NEAR/2 random):ab,ti) OR (((single OR double) NEXT/1 blind*):ab,ti) OR (((treble OR triple) NEAR/1 blind*):ab,ti) OR placebo*:ab,ti) NOT ([animals]/lim NOT [humans]/lim)

Cochrane library

(newborn:ab,ti OR neonat*:ab,ti) AND (incubator*:ab,ti OR 'infant incubator*':ab,ti OR 'neonatal incubator*':ab,ti OR 'newborn incubator*':ab,ti OR 'infant radiant warm*':ab,ti OR 'radiant heate*':ab,ti OR 'radiant warm*':ab,ti OR 'warming bed*':ab,ti OR 'warming mattress*':ab,ti OR 'heating mattress*':ab,ti OR 'warming device*':ab,ti OR 'heated water-filled mattress*':ab,ti OR thermoregulation:ab,ti OR warmer*:ab,ti OR heater*:ab,ti OR 'cot nurs*':ab,ti OR 'cot':ab,ti)

**Supplementary material 2. Risk of bias table**

Risk of bias table of included systematic reviews

| Study (systematic review) | Flenady and Woodgate 2003 | Gray and Flednady 2011 |
| --- | --- | --- |
| 1. Did the research questions and inclusion criteria for the review include the components of PICO? | Yes | Yes |
| 2. Did the report of the review contain an explicit statement that the review methods were established prior to the conduct of the review and did the report justify any significant deviations from the protocol? | No | No |
| 3. Did the review authors explain their selection of the study designs for inclusion in the review? | Yes | Yes |
| 4. Did the review authors use a comprehensive literature search strategy? | Yes | Yes |
| 5. Did the review authors perform study selection in duplicate? | Yes | Yes |
| 6. Did the review authors perform data extraction in duplicate? | Yes | Yes |
| 7. Did the review authors provide a list of excluded studies and justify the exclusions? | Yes | Yes |
| 8. Did the review authors describe the included studies in adequate detail? | Partial yes | Yes |
| 9. Did the review authors use a satisfactory technique for assessing the risk of bias (RoB) in individual studies that were included in the review? | Yes | Yes |
| 10. Did the review authors report on the sources of funding for the studies included in the review? | No | No |
| 11. If meta-analysis was performed did the review authors use appropriate methods for statistical combination of results? | Yes | Yes |
| 12. If meta-analysis was performed, did the review authors assess the potential impact of RoB in individual studies on the results of the meta-analysis or other evidence synthesis? | Yes | No |
| 13. Did the review authors account for RoB in individual studies when interpreting/ discussing the results of the review? | Yes | No |
| 14. Did the review authors provide a satisfactory explanation for, and discussion of, any heterogeneity observed in the results of the review? | Yes | Yes |
| 15. If they performed quantitative synthesis did the review authors carry out an adequate investigation of publication bias (small study bias) and discuss its likely impact on the results of the review? | Yes | Yes |

Risk of bias table of included randomised control trials

| Study (randomised control trial) | Meyer and Bold 2007 | Bhat et al. 2015 | Vijayan et al. 2020 | Chandrasekaran et al. 2021 |
| --- | --- | --- | --- | --- |
| Randomization process | Low | Low | Some concerns | Some concerns |
| Deviations from intended interventions | Some concerns | Some concerns | Some concerns | Some concerns |
| Missing outcome data | Low | Some concerns | Some concerns | Some concerns |
| Measurement of the outcome | Low | Low | Low | Low |
| Selection of the reported result | Low | Low | Low | Low |
| Overall Bias | Low | Low | Some concerns | Some concerns |

**Supplementary material 3. Guideline comparison for use of warming devices (key recommendations)**

|  | **Thermal Protection of the Newborn: A Practical Guide (WHO, 1997)** | **Managing newborn problems: a guide for doctors, nurses, and midwives (WHO, 2003)** | **WHO Recommendations on Newborn Health (WHO, 2017)** | **Nursing Manual for Neonatal Care Unit  (Cambodia, 2018)** | **Neonatal Care, Protocol for Hospital Physicians  (Egypt, 2009)** | **Neonatal Care Clinical Guidelines (Eswatini, 2018)** | **Paediatrics for Doctors (Papua New Guinea, 2003)** | **National Neonatal Protocol (Palestine, 2019)** |
| --- | --- | --- | --- | --- | --- | --- | --- | --- |
| **Prevention** | **Skin-to-skin care**  • Immediately after birth as well as later. • LBW and/or sick newborns without medical problems**.** | • Appropriate for all stable babies • Appropriate for rewarming a baby with moderate hypothermia (32°C to 36.4°C), particularly when other methods are not available.  • Not appropriate for babies with life-threatening problems (e.g. sepsis, severe breathing difficulty) | For newborns weighing 2000 g or less at birth as soon as the newborns are clinically stable. | Neonates who are stable, breathing spontaneously without additional oxygen. | • Immediately after birth as well as later. • When a neonate requires incubator care, it is important to encourage parents to visit and hold the neonate as much as possible, utilizing SSC for temperature stabilization. | Newborns without complications should be kept in SSC immediately after birth for at least an hour. | Not mentioned. | If baby is breathing or crying immediately after birth. |
|  | **Radiant warmers** For short periods for example in the delivery room, for resuscitation or during procedures in intensive care unit. This method should be replaced by other alternatives as soon as possible. | Appropriate for sick babies and babies weighing 1.5 kg or more | Unstable newborns weighing ≤ 2000 g or less at birth, or stable newborns weighing < 2000 g who cannot be given SSC**.** | Not mentioned. | • At birth, for all neonates who had low Apgar scores, exhibited signs of stress during delivery, and/or whose mothers have had prenatal and intrapartum risk factors.  • On admission to the neonatal care unit. | Unstable newborns weighing ≤ 2000 g or less at birth, or stable newborns weighing < 2000 g who cannot be given SSC**.** | For LBW babies who do not need additional oxygen. | Use a radiant warmer if room temperature is below 28°C for a small baby or twin within 90 mins from birth. |
|  | **Incubators** Incubators may be more of a hazard than a benefice to newborns if they are not used and maintained correctly. | • Appropriate for babies weighing < 1500 g who are not eligible for SSC. • Appropriate for babies who have life-threatening problems (e.g. sepsis, severe breathing difficulty) | Unstable newborns weighing ≤ 2000 g or less at birth, or stable newborns weighing < 2000 g who cannot be given SSC. | All preterm neonates ≤1500g at the admission. | During the neonatal care unit stay. | Unstable newborns weighing ≤ 2000 g or less at birth, or stable newborns weighing < 2000 g who cannot be given SSC. | Not mentioned. | Neonates ≤ 1500 g |
|  | **Heated water-filled mattresses** Safe device for keeping LBW/sick babies warm and is more economical than an incubator. The baby is kept clothed and covered with a blanket in the cot. | Not mentioned. | Not mentioned. | Not mentioned. | Not mentioned. | Not mentioned. | If SSC is not practical, use heated, water filled mattresses manufactured by Kanthal Medical Heating. | Not mentioned. |
|  | **Others** • Light-bulb heated cots or beds: Not a safe option, alternative warming methods should be used if at all possible.   • Warm rooms | Warm rooms (at least 25°C) • Appropriate for babies recovering from illness and small babies who do not require frequent diagnostic and treatment procedures. • Not appropriate for babies with life-threatening problems (e.g. sepsis, severe breathing difficulty) | Not mentioned. | Cots (cradle, open crib) with room temperature at 25-28°C for preterm neonates weighing > 1500g with a stable temperature. Covered warm bottles with cloth and put them around the neonate to avoid hypothermia if needed. | Not mentioned. | Not mentioned. | • Warm room with temperature at 27-30°C for LBW neonates.   • Small electric blankets (the Riviera 37 x 27 cm blanket) for LBW neonates. | Not mentioned. |
| **Rewarming** | **Skin-to-skin care** Mild hypothermia (body temperature 36.0-36.4°C) in a warm room at least 25°C. | Not mentioned. | Immediately after birth:  LBW neonates weighing > 1200g who do not have complications and are clinically stable immediately after birth. (Weak recommendation, low quality evidence)  Routine care:  For newborns weighing 2000 g or less at birth as soon as the newborns are clinically stable. (Strong recommendation, moderate-quality evidence). | Not mentioned. | Immediately after birth as well as later. When a neonate requires incubator care, it is important to encourage parents to visit and hold the neonate as much as possible, utilizing SSC contact for temperature stabilization. | Place the baby in SSC or in an incubator. | For LBW babies who do not need additional oxygen. | Not mentioned. |
|  | **Radiant warmers** In case of moderate hypothermia (body temperature 32-35.9°C), the clothed baby may be rewarmed under a radiant warmer. | Not mentioned. | Unstable newborns weighing ≤ 2000 g or less at birth, or stable newborns weighing < 2000 g who cannot be given SSC**.** | Warm the baby immediately using a pre-warmed infant warmer when the body temperature is < 34.9°C or prolonged hypothermia. | Use radiant warmers: (1) at birth, for all neonates who had low Apgar scores, exhibited signs of stress during delivery, and/or whose mothers have had prenatal and intrapartum risk factors, (2) on admission to the neonatal care unit. | Not mentioned. | Not mentioned. | Not mentioned. |
|  | **Incubators** In case of moderate hypothermia (body temperature 32-35.9°C), the clothed baby may be rewarmed in an incubator, at 35-36°C.  In case of severe hypothermia (body temperature below 32°C), rapid warming can be achieved using an incubator, with the air temperature set at 35-36°C. Once the baby's temperature reaches 34°C, the rewarming process should be slowed down to avoid overheating. | Not mentioned. | Unstable newborns weighing ≤ 2000 g or less at birth, or stable newborns weighing < 2000 g who cannot be given SSC. | All preterm neonates ≤ 1500g at the admission. | During the neonatal care unit stay. | Warm the baby up as quickly as possible setting the temperature to 1°C higher than the baby’s temperature, and increase as the baby warms up. Cover the baby with a plastic sheet to protect radiant heat loss. Do not cover with blankets or tin foil. Decrease the incubator temperature as the baby’s temperature returns to normal. | Not mentioned. | Incubator is better controlled than radiant warmer. Rewarm slowly (0.5˚C per hour) with a set temperature 1-1.5˚C above core temperature. |
|  | **Heated water-filled mattresses** In case of moderate hypothermia (body temperature 32-35.9°C), the clothed baby may be rewarmed by using a heated water-filled mattress.   In case of severe hypothermia (body temperature below 32°C), rapid warming can be achieved by a thermostatically controlled heated mattress set at 37-38°C. Once the baby's temperature reaches 34°C, the rewarming process should be slowed down to avoid overheating. | Not mentioned. | Not mentioned. | Not mentioned. | Not mentioned. | Not mentioned. | If SSC is not practical, use heated, water filled mattresses manufactured by Kanthal Medical Heating. | Not mentioned. |
|  | **Others** SSC, a warm room or cot if no equipment is available. | Not mentioned. | Not mentioned. | Not mentioned. | Not mentioned. | Not mentioned. | • Warm room with temperature at 27-30°C for LBW neonates.  • Small electric blankets (the Riviera 37 x 27 cm blanket) for LBW neonates. | Not mentioned. |
| **Transport** | • SSC • Full wrapping with a cap transported in the arms of an adult in a closed vehicle if SSC is not possible. • Box, padded and insulated on all side with holes for ventilation, produced locally and prewarmed in very cold weather with a hot water bottle which is removed before putting in the baby. • Transport incubators with electricity or a hot water reservoir | • Keep the baby in SSC with the mother or a relative. • During cold weather, use a warming device. If a warming device is not available, place the baby in a box with warm water bottles.  - Cover the bottles with a cloth and ensure that they are secured so that they do not directly touch the baby’s skin; - When the water becomes cold, refill the bottles with warm water or remove them from the box. | During stabilization and transfer of preterm newborns to specialized neonatal care wards, wrapping in plastic bags/wraps may be considered as an alternative to prevent hypothermia. (Conditional recommendation, low-quality evidence). | Not mentioned. | Transport incubators. | Transport incubators. | Not mentioned. | Transport incubators. |
| **Resuscitation** | Radiant heater if available | Not mentioned. | During stabilization and transfer of preterm newborns to specialized neonatal care wards, wrapping in plastic bags/wraps may be considered as an alternative to prevent hypothermia. | Use radiant warmers for short time observation or multiple procedure. Preterm infants should only be nursed on an radiant warmer in the event of multiple procedures that are unable to be undertaken in an incubator without loss of substantial heat. | Radiant warmer should be used during the performance of medical procedures. | Not mentioned. | Speedie 750w heater suspended from the ceiling on chains (it should not be mounted directly onto the ceiling) | • Preheated radiant warmers • Plastic bag or plastic wrap (< 32 weeks’ gestation) • Thermal mattress (< 32 weeks’ gestation) |
| **Strength of evidence** | Recommendations on use of devices are based on limited evidence. | Recommendations are based on the latest available scientific evidence (not cited) and the guide. | Recommendations are based on the Grading of Recommendations, Assessment, Development and Evaluation (GRADE) approach. | Recommendations are based on other guidelines and protocols. | Recommendations are based on other guidelines, protocols and previous studies. | Recommendations are based on other guidelines and protocols. | Recommendations are based on previous research including one randomised control trial. | Recommendations are based on other guidelines, protocols and previous studies. |

LBW = Low birth weight

SSC = Skin-to-skin care
